# Free-breathing half-radial dual-echo balanced steady-state free precession thoracic imaging with wobbling Archimedean spiral pole trajectories

**DOI:** 10.1016/j.zemedi.2022.01.003

**Published:** 2022-02-18

**Authors:** Oliver Bieri, Orso Pusterla, Grzegorz Bauman

**Affiliations:** a Division of Radiological Physics, Department of Radiology, University of Basel Hospital, Basel, Switzerland; b Department of Biomedical Engineering, University of Basel, Basel, Switzerland

**Keywords:** Radial MRI, Non-Cartesian trajectory, Free-breathing, Balanced steady-state free precession, TrueFISP, Lung imaging

## Abstract

**Purpose:**

To demonstrate free-breathing thoracic MRI with a minimal-TR balanced steady-state free precession (bSSFP) technique using wobbling Archimedean spiral pole (WASP) trajectories.

**Methods:**

Phantom and free-breathing *in vivo* chest imaging in healthy volunteers was performed at 1.5T with a half-radial, dual-echo, bSSFP sequence, termed bSTAR. For maximum sampling efficiency, a single analog-to-digital converter window along the full bipolar readout was used. To ensure a homogeneous coverage of the k-space over multiple breathing cycles, radial k-space sampling followed short-duration Archimedean spiral interleaves that were randomly titled by a small polar angle and rotated by a golden angle about the polar axis; depticting a wobbling Archimedean spiral pole (WASP) trajectory. In phantom and *in vivo* experiments, WASP trajectories were compared to spiral phyllotaxis sampling in terms of eddy currents and were used to generate *in vivo* thorax images at different respiratory phases.

**Results:**

WASP trajectories provided artifact-free bSTAR imaging in both phantom and *in vivo* and respiratory self-gated reconstruction was successfully performed in all subjects. The amount of the acquired data allowed the reconstruction of 10 volumes at different respiratory levels with isotropic resolution of 1.77 mm from a scan of 5.5 minutes (using a TR of 1.32ms), and one high-resolution 1.16 mm end-expiratory volume from a scan of 4.7 minutes (using a TR of 1.42ms). The very short TR of bSTAR mitigated off-resonance artifacts despite the large field-of-view.

**Conclusion:**

We have demonstrated the feasibility of high-resolution free-breathing thoracic imaging with bSTAR using the wobbling Archimedean spiral pole in healthy subjects at 1.5T.

## 1 Introduction

Despite the continuous improvement in MRI hardware and data processing techniques, thoracic MRI remains challenging due to the problems associated with several physical properties of the lung [1]. The main problem arises from the foam-like structure of the lung parenchyma resulting in a proton density that is much lower compared to the surrounding tissues. Moreover, strong magnetic susceptibility differences in the lung caused by a large number of air-tissue and air-liquid interfaces result in extremely short transverse relaxation times (T2* of 1-2ms and T2 of 30-80ms at 1.5T), while the longitudinal relaxation time (T1) of 1000-1500ms is relatively long [2], [3], [4]. Moreover, respiratory and cardiac motion as well as pulsation and blood flow, complicate image acquisition. Overall, signal-to-noise ratio (SNR) of the lung is poor and MRI can be further degraded by the presence of motion artifacts.

Nevertheless, during the past two decades, a significant amount of effort has been made to develop MRI techniques for the assessment of lung structure. Imaging techniques based on ultra-short echo time (UTE) [5], [6], zero echo time (ZTE) [7], [8] have shown compelling results for thoracic imaging at 1.5T and 3T. Both techniques allow for improved detection of the fast decaying signal in tissues with short T2*. Three-dimensional (3D) high-resolution (∼1 mm) UTE or ZTE acquisitions require several minutes of scan time exceeding an average breath-hold time limit and thus must be combined either with gating or self-gating techniques [6], [9], [10]. In the recent years the application of UTE for structural and functional lung imaging has been extensively explored [11].

Another technique allowing for substantial increase in SNR of the lung tissue is balanced steady-state free precession (bSSFP) imaging [12], [13]; a pulse sequence that is highly valued in cardiovascular MRI [14], [15], [16], [17], [18], [19]. As a rapid imaging technique, bSSFP offers a unique T2/T1 contrast, highest SNR per time unit compared to incoherent SSFP pulse sequences [20], and is generally motion insensitive [21]. Despite its increasing popularity, even on the modern clinical MR-systems, bSSFP imaging can be technically demanding. The technique is very sensitive to any source of imperfection that can perturb the perfectly balanced series of gradient pulses [22]. Furthermore, off-resonances lead to periodic modulation of the steady-state having high intensity signal regions, frequently referred to as “pass-band” regions, and so-called “stop-bands” or “banding artifacts”, where signal intensity comes close to zero. Generally, banding artifacts can be mitigated by a shortening of the repetition time (TR). It was demonstrated that with optimized RF pulse and gradient timings at TR of 3D Cartesian bSSFP can be pushed close to 1ms providing remarkably increased signal in the lung parenchyma and isotropic resolution of about 1.8–2.4 mm [23], [24].

In order to improve the robustness against motion and to increase the sampling efficiency, while keeping minimal TR, a 3D radial bSSFP acquisition technique termed bSTAR has been recently proposed [25]. The technique employs dual-echo half-radial sampling by using bipolar gradients and non-selective radio frequency excitations separated by minimum hardware specific transmit/receive switching time constraints. Consequently, the k-space is sampled with both center-out and center-in half-radial projections. A similar bSSFP-based imaging approach was presented by Lu et al [26] in form of a bowtie-like readout, and more recently by Diwoky et al [27]. Thoracic imaging with bSTAR has demonstrated to provide high-resolution and artifact-free acquisitions obtained during a single breath-hold allowing for an improved visualization of pulmonary parenchyma and vascular tree. However, the requirement for a prolonged breath-hold limits the feasibility of the technique especially in children, uncooperative patients, or patients with severe pulmonary disease. An important prerequisite for a free-breathing imaging is to ensure a homogeneous coverage of the k-space over multiple breathing cycles during a breathing phase of interest. In contrast to the 3D radial imaging with spoiled gradient echo (SPGR), eddy-currents artifacts pose a serious challenge for the bSSFP-based acquisitions [22]. Approaches successfully employed for 3D lung UTE imaging i.e. using a k-space trajectory with pseudo-random reordering [6], cannot be combined with a bSSFP kernel since the large jumps in k-space can induce eddy-currents and lead to a severe image quality deterioration [28]. Hence, the k-space trajectory has to be carefully designed. For cardio-vascular MRI, a solution to this problem was recently proposed using a spiral phyllotaxis trajectory [28], which allows sampling of the k-space along a smooth trajectory for free-running 3D radial bSSFP sequences. However, the spiral phyllotaxis generates non-smooth trajectories for interleave numbers that do not belong to the Fibonacci series. Furthermore, the spiral phyllotaxis trajectory is characterized by an increased non-uniformity in the distribution of radial readouts in spherical coordinates in comparison to trajectories based on the uniform Archimedean spiral.

In this work we present the concept of a wobbling Archimedean spiral pole (WASP) trajectory that generates smooth trajectories and uniform distributions of radial readouts. The feasibility of the free-breathing bSTAR was tested in a phantom and a group of healthy volunteers

## 2 Materials and methods

### 2.1 Free-breathing bSTAR

**Figure 1:**
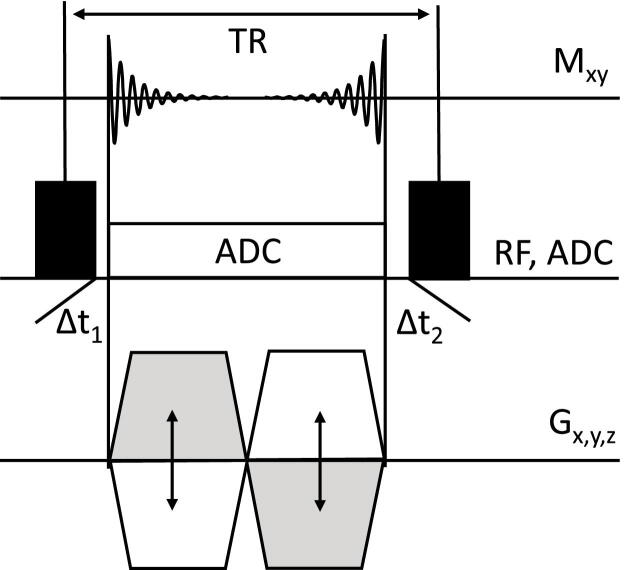
Pulse sequence diagram for bSTAR imaging featuring half-radial dual-echo readout along the bipolar gradients G_x_, G_y_, G_z_ together with a non-selective radio frequency (RF) excitation pulse. One single analog-to-digital converter (ADC) window is used that spans over the full bipolar gradient moment, thus offering maximum pulse sequence efficiency. The time delay Δt_1_ (between the RF and the start of the ADC) was set to 30 μs allowing for coil ring-down, whereas time delay Δt_2_ (between the ADC and the consecutive RF excitation) was 110 μs.

We have implemented a 3D half-radial dual-echo acquisition scheme using a bSSFP kernel with a non-selective rectangular radio-frequency (RF) excitation pulse and a bipolar readout gradient similarly to previously presented techniques [25], [27]. In contrast to prior work, however, the signal was sampled along the full bipolar radial readout gradient (i.e., including all ramps). As a result, a single analog-to-digital converter (ADC) window was used, the sampling efficiency (defined as the fraction of the TR that is used for sampling the signal) is maximized and the spatial resolution is improved. This holds especially for short TR and high-band bandwidth acquisitions where the gradient ramp times constitute a large fraction of the bipolar gradient time length. The diagram of the pulse sequence is presented in Figure 1.

To ensure a homogeneous coverage of the k-space, we have applied rapidly interleaved Archimedean spiral trajectories [29] rotated about the polar axis by the golden angle $\psi_{gold}=\pi \left(3+\sqrt{5}\right)$ [30]. In order to further mitigate the sampling periodicity with respect to the breathing cycle, each consecutive Archimedean spiral interleaf is titled by a small random polar angle with a floating point precision in range of 0° to 10° that is a wobbling Archimedean spiral pole (WASP). Any lack of tilting of the consecutive interleaves or a very low tilting angle ranges results in an inadequate k-space coverage and undersampling near the polar regions. For mitigation of eddy-currents, it is essential to avoid large jumps in the k-space [22]. Thus, consecutive spiral interleaves alternate the direction in which they traverse the k-space along the azimuthal axis by following a smooth trajectory. In this way each consecutive interleaf starts near the last half-radial projection of the previous interleaf. A preparing pulse scheme consisting of an α/2 RF pulse [31] followed by 500 dummy TRs with a constant α RF pulse was used prior to the data acquisition to enhance the transition into the steady-state.

**Figure 2:**
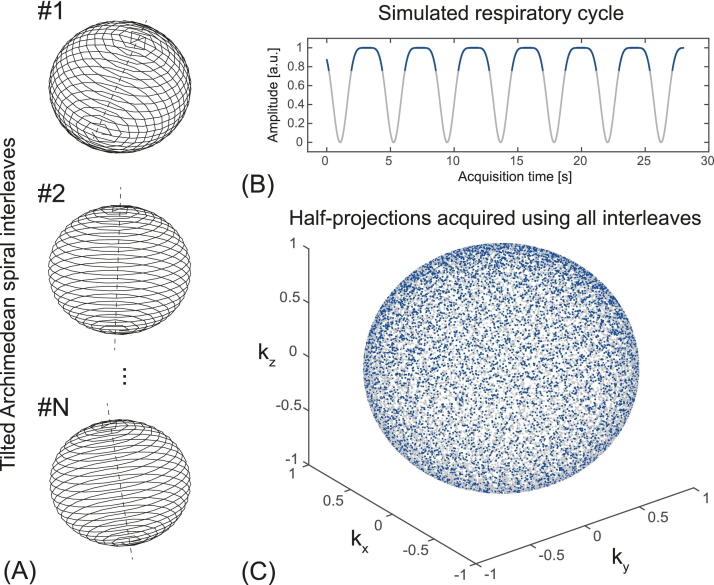
K-space sampling strategy using wobbling Archimedean spiral pole (WASP) trajectoryfor free-breathing bSTAR acquisition. For illustration purposes the k-space trajectory was simulated using 40’000 half-radial projections and N = 80 randomly tilted interleaves. The center-out and center-in half-radial projections follow an Archimedean trajectory in spherical coordinates. Each consecutive Archimedean spiral interleaf is tilted by random polar angle in range of 0° to 10° and rotated by a golden angle about the polar axis (A). The breathing cycle was simulated using the Lujan formula (33): $s\left(t\right)=1.0-cos^{2n}\left(\pi t/T-\varphi \right)$ with respiratory cycle period T=3s, n=3 and φ=π/3 (B). Blue and gray dots on the plot (C) represent all acquired half-radial projections during the acquisition. Blue dots accounting for approximately 40% of the data samples, were acquired during the expiratory phase of the breathing cycle marked with blue line.

Figure 2 illustrates the simulated k-space sampling strategy based on WASP trajectory acquired during free-breathing. The respiratory cycle was simulated using the Lujan formula (Eq. 2 in [32]) taking into account shorter inspiration than expiration times for normal breathing (I/E ratio of approximately ½ [33]).

### 2.2 MRI data acquisitions

MRI experiments were performed on a 1.5T whole-body scanner (MAGNETOM Avanto-Fit, Siemens Healthineers, Erlangen, Germany) using 12-channel thorax and 24-channel spine coils for signal reception. All scans were performed in isocenter with predefined default shim settings (i.e. in tune up mode). An accredited American College of Radiology (ACR) structural phantom was scanned using bSTAR with the WASP trajectory, as well as the spiral phyllotaxis trajectory, adapted for half-radial projections [34]. The dimension of the phantom was 14.8 cm in length and 19 cm in diameter. The acrylic plastic hollow cylinder was filled with a 10 mM NiCl2 and 75 mM NaCl solution. For *in vivo* experiments five healthy volunteers (mean age: 36.6 years, range 27-53 years, three male, two female) were scanned in supine position with the free-breathing bSTAR and a single expiratory breath-hold bSTAR for comparison. In one volunteer free-breathing bSTAR scans were performed using both, i.e. the WASP and spiral phyllotaxis trajectories. The study was approved by the Institutional Review Board, and written informed consent was obtained from each volunteer prior to the examinations.

The acquisitions in the phantom were performed with the following parameters: FOV = 32cm×32cm×32 cm, twofold readout oversampling, TE_1_/TE_2_/TR = 0.11/1.65/1.84ms, 0.98 mm isotropic resolution, 384 samples per half-radial projection, 150 μs hard RF pulse, flip angle α = 20°, 1302 Hz/pixel bandwidth, acquisition time of 57s. Four different trajectory setups were applied: WASP trajectory using 88 and 89 interleaves with 31’152 and 31’150 half-radial projections, as well as the spiral phyllotaxis trajectory using 88 and 89 interleaves with 31’152 and 31’150 half-radial projections, respectively.

*In vivo* bSTAR scans with the WASP trajectory were performed with the following pulse sequence parameters: FOV = 35cm×35cm×35 cm, twofold readout oversampling. Two different bSTAR parameter setups were used. The first bSTAR setup aimed for a multi-volumetric acquisition and the single breath-hold bSTAR acquisition was performed with the following parameters: TE_1_/TE_2_/TR = 0.11/1.13/1.32 ms, 1.77 mm isotropic resolution, 256 samples per half-radial projection, 150 μs hard RF pulse, flip angle α = 20°, 2056 Hz/pixel bandwidth. For the free-breathing bSTAR 250’000 half-radial projections with 500 interleaves were used and resulted in 5.5 min scan time (0.66s per interleaf). The breath-hold bSTAR scan was acquired using 17’000 half-radial projections and 12 interleaves in 23s. The pulse sequence efficiency was 0.78 for both acquisitions. The second parameter setup for the free-breathing bSTAR acquisition was tuned for high-resolution reconstruction of only one expiratory phase. The imaging parameters were as follows: TE_1_/TE_2_/TR = 0.08/1.26/1.42ms, 1.16 mm isotropic resolution, 416 samples per half-radial projection, 100 μs hard RF pulse, flip angle α = 20°, 1717 Hz/pixel bandwidth, sequence efficiency of 0.83, 200’000 half-radial projections and 400 interleaves. The scan resulted in 4.7 min acquisition time (0.71s per interleaf). The acquisitions were performed using the WASP trajectory. In order to correct for the discrepancy between the nominal and actual k-space trajectory of the bSTAR acquisition, a short calibration scan of 20s was performed once for each parameter setup. The calibration was performed by measuring the phase progression resulting from readout gradients oriented along each axis separately as proposed by Duyn et al [35]. The actual k-space trajectory corresponds to the spatial variation in the resonant frequency and the phase progression introduced by the gradient along given axis.

### 2.3 Image reconstruction and retrospective self-gating

The acquired bSTAR data were reconstructed off-line. For the self-gated free-breathing scans the respiratory signal modulation in the k-space center was used as described previously [36]. To extract the respiratory signal from the signal modulations picked up by the chest and spine phased arrays we used randomized singular value decomposition (rSVD) [37]. The extracted signal was used to sort the readouts into bins corresponding to different phases of the respiratory cycle.

All scans were reconstructed using compressed sensing with a fast iterative shrinkage-thresholding algorithm [38]. The data acquired at both echoes (i.e. obtained from the center-out and center-in half-radial projection) was reconstructed separately and added in the complex space after the iterative reconstruction process. The phantom scans were reconstructed on a 320^3^ matrix with 1.0 mm isotropic resolution. The breath-hold and free-breathing bSTAR scan using setup 1, were reconstructed on a 384^3^ matrix with 256 samples per half-spoke resulting in 1.77 mm isotropic resolution interpolated to 1.4 mm. The readouts were sorted into 10 bins corresponding to different respiratory phases. The reconstruction time of a multi-volume dataset took approximately 24 minutes.

The free-breathing bSTAR scan using setup 2, was reconstructed on 512^3^ matrix with 416 samples per half-spoke using 25% of the data acquired in expiratory phase of the breathing cycle and resulting in isotropic resolution of 1.16 mm matching the resolution of the acquired k-space data. The reconstruction of the high-resolution dataset took approximately 6 minutes. Furthermore, maximum intensity projection (MIP) images were calculated for visualization of the pulmonary vasculature.

The software for off-line image reconstruction was written in C++ as stand-alone (GNU Compiler Collection 11.2 64-bit on Linux operating system) and CUDA Toolkit 11.4 (NVIDIA Corp., Santa Clara, CA). The reconstruction workstation was equipped with 2x AMD Epyc 7502 CPU (AMD Inc., Santa Clara, CA) and NVIDIA Quadro RTX 8000 GPU (NVIDIA Corp.).

## 3 Results

**Figure 3:**
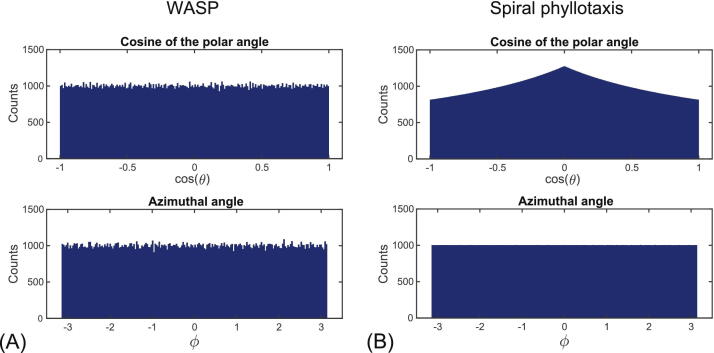
Histograms showing the cosine of the polar angle and the azimuthal angle distributions of half-radial projections generated for bSTAR with (A) the wobbling Archimedean spiral pole (WASP) trajectory with 200’000 projections and 400 interleaves, and (B) phyllotaxis trajectory with 199’810 and 377 interleaves. The distribution of the cosine of the polar angle and the azimuthal angle of the half-radial projections were used to assess the spatial homogeneity of the k-space trajectory. The WASP trajectory provides uniform distributions in both angular dimensions with the expected noise-like component caused by randomly tilting the spiral interleaves. The distribution of the azimuthal angle for the spiral phyllotaxis trajectory is perfectly uniform. It is however not uniform for the cosine of the polar angle, and thus a higher sampling density near the equatorial plane is expected.

Figure 3 presents histograms showing the cosine of the polar angle and azimuthal angle distributions of half-radial projections generated for a WASP and a phyllotaxis trajectory as a measure of homogeneity of data sampling in spherical coordinates. WASP trajectories are characterized by uniform distribution of half-radial projections. However, a higher sampling density near the equatorial plane is expected when using the phyllotaxis trajectory.

**Figure 4:**
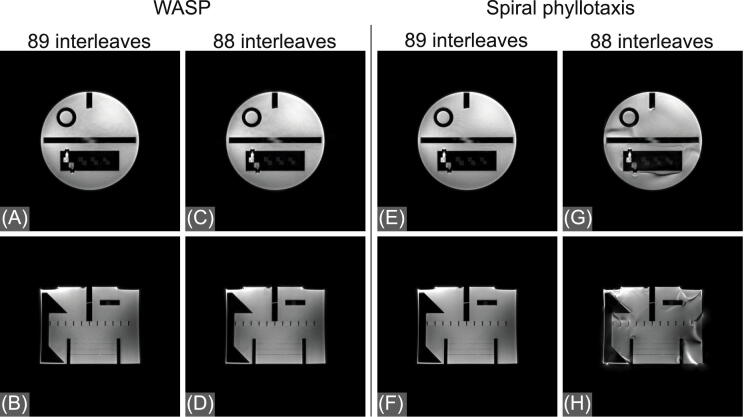
A comparison between coronal and sagittal images obtained in a structural phantom using bSTAR with the wobbling Archimedean spiral pole (WASP) trajectory and the spiral phyllotaxis trajectory. The spiral phyllotaxis trajectory acquisition with the number of interleaves different than a Fibonacci number (Fig. 4G and 4H) is severely affected by eddy currents artifacts.

**Figure 5:**
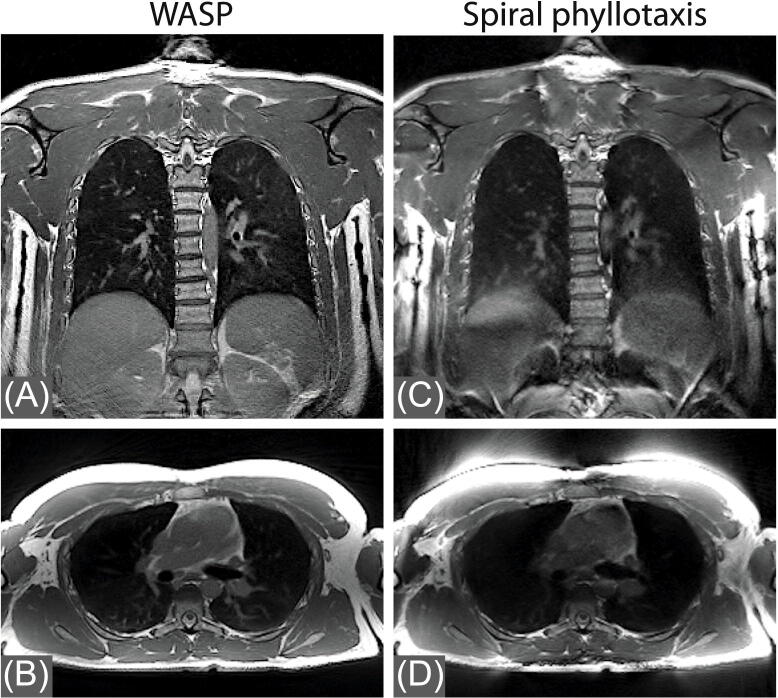
Comparison between coronal and sagittal *in vivo* images obtained in healthy volunteer #2 using free-breathing bSTAR with the wobbling Archimedean spiral pole (WASP) trajectory (A,B) and the spiral phyllotaxis trajectory (C,D). Both scans were performed using setup 2 with 200’000 half-radial projections and 400 interleaves.

Coronal and sagittal images obtained in the structural phantom using bSTAR with the WASP and the spiral phyllotaxis trajectories are shown in Figure 4. The image quality of the bSTAR scans using WASP is unaffected by the number of the interleaves used. However, this does not hold for the bSTAR scans with the spiral phyllotaxis trajectory since any deviation from the Fibonacci number of interleaves results in non-smooth trajectories introducing severe eddy currents artifacts (Fig. 4G and 4H). A comparison between *in vivo* images acquired using free-breathing bSTAR with the WASP and the spiral phyllotaxis trajectory with 400 interleaves is shown in Figure 5. Similarly to the phantom experiment, a non-Fibonacci number for the number of interleaves for the spiral phyllotaxis results in severe degradation of image quality caused by eddy currents.

**Figure 6:**
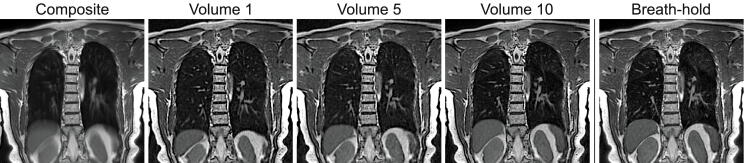
Illustrative coronal images (1.77 mm isotropic resolution interpolated to 1.4 mm) acquired using setup 1 of the free-breathing bSTAR with the wobbling Archimedean spiral pole (WASP) trajectory in the volunteer #1: using all acquired data (no gating) - composite, data binned to end inspiration - volume 1, data binned to intermediate respiratory state - volume 5 and data binned to end expiration - volume 10. For comparison breath-hold bSTAR acquisition was performed. All ten reconstructed volumes are shown in Supplementary Material Figure 1.

**Figure 7:**
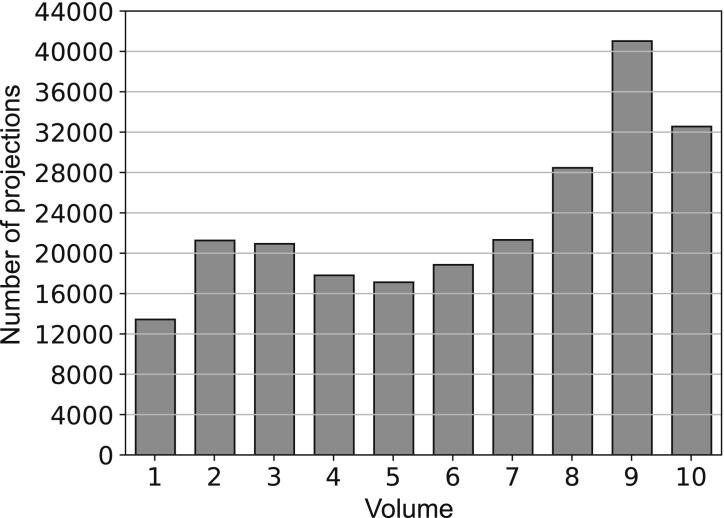
Histogram showing the number of half-radial projections used for the reconstruction of the multivolume dataset presented in Figure 6 and in Supplementary Material Figure 1 with the volume 1 and 10 at the end-inspiratory and end-expiratory phases respectively.

**Figure 8:**
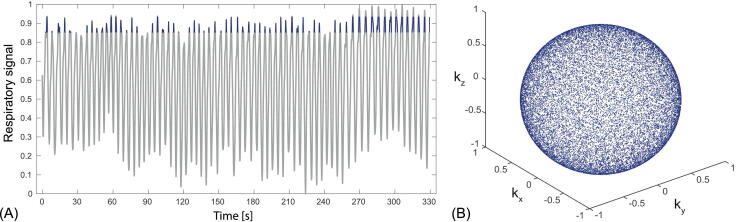
Diagram (A) shows the respiratory signal extracted from the dataset presented in Figure 6 as well as a 3D plot (B) visualizing half-radial projections used for the reconstruction of volume 10 as blue dots on a sphere. The data used for the reconstruction of volume 10 were marked blue on the respiratory signal timecourse. Data corresponding to values of the respiratory time-course below the 0.05 quantile and above the 0.95 quantile were discarded as outliers during the reconstruction.

Coronal images reconstructed at different respiratory levels, from a healthy volunteer using free-breathing bSTAR with the WASP trajectory (setup 1) are shown in Figure 6. For comparison, a single breath-hold bSTAR reconstruction is also given. As expected, the reconstruction without self-gating shows heavily blurred pulmonary morphology; especially in the lower airways. The self-gated reconstruction was used to generate 10 volumes with interpolated 1.4 mm isotropic resolution at different respiratory levels, allowing for markedly improved visualization of pulmonary and vascular structures. The histogram in Figure 7 shows the number of half-radial projections that were used for the reconstruction of the different respiratory phase. In tidal breathing most of the data was acquired during the expiratory phase consequently providing the best k-space coverage in comparison to other respiratory phases. Interestingly, the high signal intensity in the lung parenchyma provided by bSSFP enabled us to observe differences in signal intensity levels between lung lobes, which are particularly visible during the expiratory phase. Overall, visually, no difference is observed between the end-expiratory free-breathing bSTAR with WASP trajectory and the single breath-hold bSTAR end-expiratory acquisition. Images showing slices from all ten reconstructed volumes are presented in Supplementary Material Figure 1. The extracted respiratory signal from this dataset, the data range used for the reconstruction of volume 10 as well as a 3D plot showing the resulting k-space coverage is shown in Figure 8.

**Figure 9:**
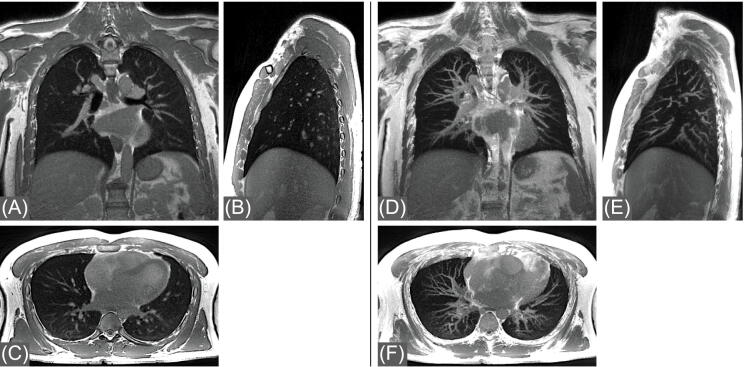
Coronal (A), sagittal (B) and axial (C) chest images acquired using free-breathing bSTAR with the wobbling Archimedean spiral pole (WASP) trajectory in the healthy volunteer #1 with 1.16 mm isotropic resolution and reconstructed using 25% of data measured in the expiratory phase. Maximum intensity projection image reconstructions (D,E,F) were performed with 20 mm thickness at corresponding slice locations.

Figure 9 shows coronal, sagittal and axial end-expiratory images reconstructed from the free-breathing bSTAR WASP data (setup 2) acquired in a healthy volunteer with 1.16 mm isotropic resolution. The high spatial resolution resolves fine pulmonary structures such as peripheral vessels which can be especially well appreciated in the corresponding 20 mm maximum intensity projection (MIP) reconstructions. The high steady-state signal of blood for bSSFP allows a detailed visualization of the pulmonary vasculature without the use of intravenous contrast agent. Overall, no banding artifacts crossing the pulmonary vasculature or artifacts caused by cardiac motion were observed. Example images obtained from the free-breathing bSTAR WASP data from the remaining three volunteers are shown in the Supplementary Material Figure 2.

## 4 Discussion

In this work, we have introduced a free-breathing bSSFP imaging technique for the assessment of thoracic morphology. The presented technique employs a minimal-TR bSSFP kernel with dual-echo half-radial readout as previously introduced for breath-hold chest imaging at 1.5T [25]. In addition, the pulse sequence's kernel was optimized by using one single ADC spanned along the full bipolar readout gradient moment. For equal TR, this offers a spatial improvement of about 10-15% compared to the pulse sequence implementation in the previous work; particularly for high-bandwidth acquisitions where the gradient ramp times constitute a large fraction of the bipolar readout gradient moment.

The major technical improvement presented in this work, is the introduction of a smooth and rapidly interleaved k-space trajectory following randomly tilted Archimedean spirals in spherical coordinates allowing for eddy-current artifacts-free bSSFP imaging. For homogenous k-space coverage during free-breathing and mitigation of the synchronicity with the respiratory cycle the consecutive short-duration interleaves were rotated with a golden angle increment and tilted by a small random angle along the azimuthal axis. The proposed k-space sampling strategy offers an increased flexibility in choosing the duration and number of interleaves in comparison to previously presented solutions such as the spiral phyllotaxis trajectory. Moreover, the wobbling Archimedean spiral pole trajectory offers a near uniform distribution of half-radial projections on the sphere consequently allowing for the application of simple density compensation during image reconstruction.

Generally, bSSFP provides higher signal intensity per unit time than SPGR and the dual-echo sampling of rapidly decaying T2* species is especially beneficial for bSSFP since the both echoes acquired directly before and after the RF excitation have almost equal amplitude [25]. As a result, bSTAR imaging offers not only an improved visualization of the vascular structure in comparison to the contemporary imaging techniques but also a boost in the parenchymal signal of up to 4 times in comparison to a standard UTE acquisitions [25]. Furthermore, the very short TR of bSTAR on the one hand mitigates problems related to off-resonances [39] and on the other hand offers about 2 to 3 times more radial projections per unit time, offering reduced undersampling effects in comparison to contemporary UTE lung imaging parameter settings [6]. From the current setting, bSTAR with the WASP trajectory offers 1.16 mm isotropic resolution in one expiratory volume with a 4.7 min free-breathing scan which is generally well-acceptable in the clinical setting [40]. A single 5.5 min free-breathing bSTAR dataset acquired with the WASP trajectory allowed for a multi-volumetric reconstruction of up to 10 volumes at different respiratory phases with high-quality isotropic resolution of 1.77 mm. Despite rapid acquisition with short TR for several minutes, the specific absorption rate and gradient stimulation values were kept below the required safety limit.

Respiratory self-gating addresses a major limitation of the previously presented half-radial dual-echo 3D bSSFP imaging approach [25]. The possibility of performing free-breathing acquisitions is especially important for patients with advanced respiratory disease, uncooperative patients or children. A possible limitation of our study is the facts that the respiratory self-gated reconstruction was tested only in healthy subjects with normal breathing patterns. Previous studies, however, have shown similar respiratory self-gating techniques in patients with pulmonary disease [6], [8], [9], [10]. Nevertheless, the performance of free-breathing bSTAR and its usefulness to assess thoracic morphology or pathological changes in patients with pulmonary disease has yet to be tested in future studies.

## 5 Conclusions

In this work, we have demonstrated feasibility of respiratory self-gated free-breathing 3D bSTAR imaging in healthy human subjects. In order to mitigate eddy current artifacts and achieve homogeneous k-space coverage during free-breathing we proposed a k-space sampling strategy based on the wobbling Archimedean spiral pole trajectory. The ability to perform free-breathing scans represents an important technical improvement of bSTAR imaging and is especially important for less compliant subjects. Future studies are required to evaluate the performance of this technique for the assessment of lung morphology in patients with pulmonary disease.

## 6 Declaration of interests

The authors declare that they have no known competing financial interests or personal relationships that could have appeared to influence the work reported in this paper.

The authors declare the following financial interests/personal relationships which may be considered as potential competing interests:
