# Erratum to “Free-breathing half-radial dual-echo balanced steady-state free precession thoracic imaging with wobbling Archimedean spiral pole trajectories” [Z Med. Phys. 33 (2023) 220–229]

**DOI:** 10.1016/j.zemedi.2024.07.003

**Published:** 2024-09-02

**Authors:** Oliver Bieri, Orso Pusterla, Grzegorz Bauman

**Affiliations:** a Division of Radiological Physics, Department of Radiology, University of Basel Hospital, Basel, Switzerland; b Department of Biomedical Engineering, University of Basel, Basel, Switzerland

The publisher regrets that the declaration of competing interest statement was not included in the original article. The correct declaration of competing interest statement for this article is:

The authors declare that they have no known competing financial interests or personal relationships that could have appeared to influence the work reported in this paper.

The publisher would like to apologise for any inconvenience caused.

