# Supplementary material for: Free-breathing half-radial dual-echo balanced steady-state free precession thoracic imaging with wobbling Archimedean spiral pole trajectories
Source: Z Med Phys. 2022 Feb 18;33(2):220–9. doi: 10.1016/j.zemedi.2022.01.003 (PMC10311259; doi:10.1016/j.zemedi.2022.01.003)
Supplement: Supplementary file 1 [file mmc1.pdf]

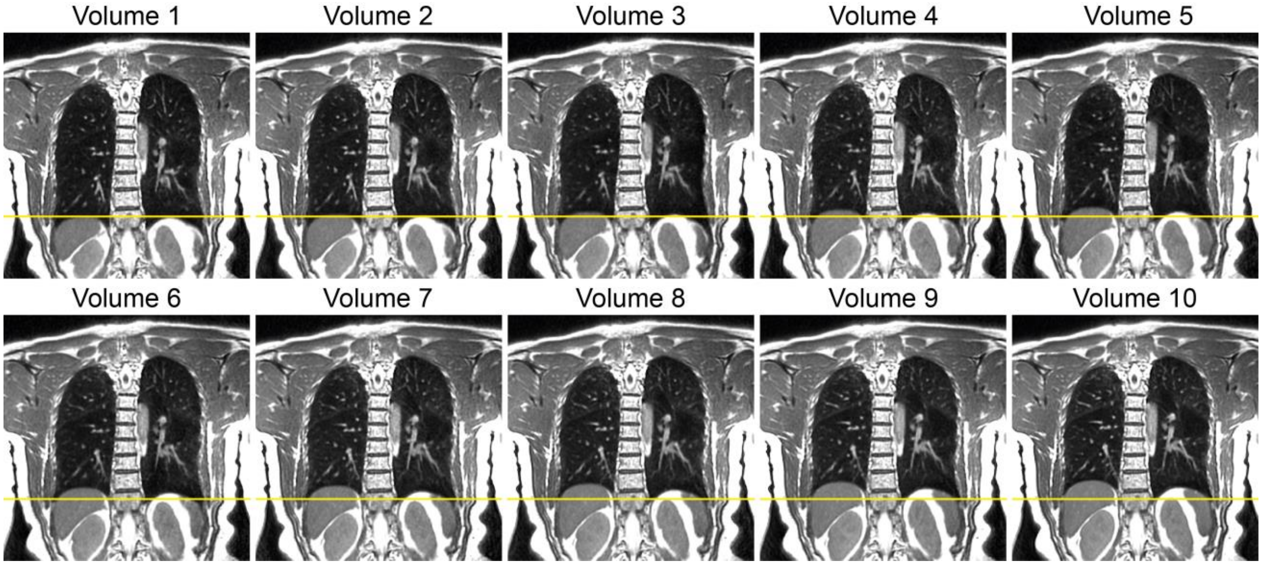

Supplementary Material Figure 1. Coronal chest images obtained in a healthy volunteer from ten volumes reconstructed from a single free-breathing bSTAR with WASP trajectory (1.77mm isotropic resolution interpolated to 1.4mm) using setup 1. The yellow line indicates the position of the diaphragm during the end-inspiratory phase (volume 1).
