# Supplementary material for: Free-breathing half-radial dual-echo balanced steady-state free precession thoracic imaging with wobbling Archimedean spiral pole trajectories
Source: Z Med Phys. 2022 Feb 18;33(2):220–9. doi: 10.1016/j.zemedi.2022.01.003 (PMC10311259; doi:10.1016/j.zemedi.2022.01.003)
Supplement: Supplementary file 2 [file mmc2.pdf]

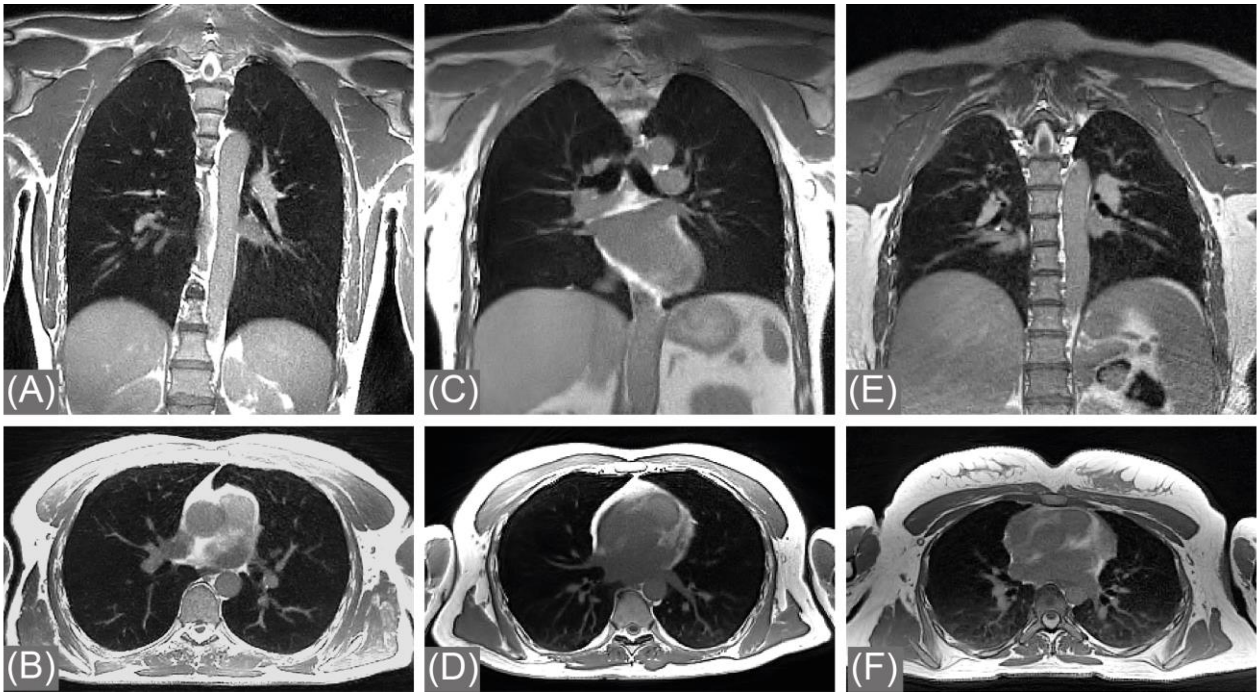

Supplementary Material Figure 2. Exemplary coronal and sagittal images obtained in volunteers #3 (A,B), #4 (C,D) and #5 (E,F) using free-breathing bSTAR with WASP trajectory (setup 1).
